# Reply to Akça, B.; Bilotta, F. Time and Type of Administered Fluids during Cesarean Section Might Not Matter for Hemodynamic Outcomes, but There Are Significant Patient Safety Concerns Regarding Colloid Use in Parturients. Comment on “Theodoraki et al. Colloid Preload versus Crystalloid Co-Load in the Setting of Norepinephrine Infusion during Cesarean Section: Time and Type of Administered Fluids Do Not Matter. *J. Clin. Med.* 2023, *12*, 1333*”*

**DOI:** 10.3390/jcm12144754

**Published:** 2023-07-18

**Authors:** Kassiani Theodoraki, Sofia Hadzilia, Dimitrios Valsamidis, Konstantina Kalopita, Emmanouil Stamatakis

**Affiliations:** 1 Department of Anesthesiology, Aretaieion University Hospital, National and Kapodistrian University of Athens, 11528 Athens, Greece; 2 Department of Anesthesiology, Alexandra General Hospital of Athens, 11528 Athens, Greece

We would like to take this opportunity to thank Drs Akça and Bilotta for their interest and their insightful comments [1] concerning our work [2]. In response to the issues raised, we would like to note the following facts:

1. Regarding the first comment about the fact that intraoperative use of hydroxyethylstarch (HES) remains controversial, we have highlighted in our original publication that colloid administration can be complicated by untoward effects such as anaphylactoid reactions and compromise of renal function in high-risk patients. We have also pointed out the recent warning by the U.S. Food and Drug Administration and the European Medicines Agency regarding the use of colloids in sepsis, kidney impairment and critically ill patients. This is the reason we proposed the solution of crystalloid co-loading as a viable and less costly alternative strategy in the context of maternal hypotension. However, as Drs Akça and Bilotta correctly point out, there are still open questions as to the generalizability of data regarding precautions of colloid use and it remains unclear whether the same risks as those identified in high-risk patients exist in the routine perioperative setting (such as in cases of Cesarean delivery, where the majority of parturients do not have underlying comorbidities). This is the reason why in the consensus about the management of maternal hypotension by Kinsella et al., colloid pre-loading and co-loading are suggested as recommendations of best clinical practice along with crystalloid co-loading for the mitigation of spinal-induced hypotension [3]. Additionally, the most recent metanalysis published on fluid loading therapy to prevent spinal hypotension in women undergoing Cesarean section, supported the efficacy of colloid pre-or co-loading and to a lesser extent of crystalloid co-loading for decreasing the incidence of hypotension [4]. Furthermore, even in the reference provided by Drs Akça and Bilotta [5], the authors themselves acknowledge that there is no evidence to support total suspension of HES, even in critically ill patients, providing evidence about it from large trials and a metanalysis [6,7,8]. Therefore, we believe that HES use especially in contexts of rationalized use, such as in cases of maternal hypotension, should continue. In light of this, De Hert et al. also stressed that when used with the proper indication, and considering the recommended doses, HES still has a place in perioperative fluid management [9].

2. Regarding the second comment raised by Drs Akça and Bilotta, we definitely agree that spinal-induced maternal hypotension should be treated or prevented preferentially by vasopressors, since the main pathophysiologic mechanism underlying maternal hypotension is the decrease in sympathetic tone in the arterial vascular network [10]. This is properly pointed out in our original publication, where we put emphasis on the fact that the main strategy for vascular tone maintenance in the obstetric anesthesia setting is the administration of a vasoactive agent. However, fluid administration continues to be used by many obstetric anesthesiologists in the algorithm of hypotension management [4] and in fact, combination techniques seem to be more effective than single interventions, as suggested by recent network metanalyses [4,11]. In fact, there is literature evidence that there is a higher incidence of hypotension when a vasoactive agent is administered alone without the simultaneous administration of fluids [12]. In our study, we did not administer HES preemptively s a sole agent but in combination with a continuous infusion of the vasopressor agent norepinephrine, since the rationale for our protocol was to check whether colloid preload would be superior to crystalloid co-load in the setting of a background norepinephrine infusion.

3. As to the third issue raised by the authors, we would like to note that in the publication by Akay et al., mentioned by Drs Akça and Bilotta [13], the population investigated was totally different to ours, consisting of 22 women with gynecologic malignancies. Pregnancy is well-known to be associated with a hypercoagulable state and the changes occurring to the coagulation system of the parturient lead to approximately double the coagulation activity seen when compared with the non-pregnant state, a fact highlighted in the second publication mentioned by Drs Akça and Bilotta in relation to this matter [14]. These coagulation changes in combination with inferior vena cava compression by the gravid uterus promote thrombosis rather than hemorrhage [15,16,17]. In fact, the aforementioned alterations and the associated prothrombotic state may actually make neuraxial procedure and other invasive interventions safer in parturients than in other patients [16]. In another study, comparing the effects on thrombelastography (TEG) of preloading with two different colloid fluids prior to spinal anesthesia for Cesarean section, it was demonstrated that all TEG parameters remained within or very close to the normal reference range in both colloid groups [18]. It has been recommended, however, that the maximum dose of HES 130/0.4 should not exceed 15–20 mL/kg, a limit which by no means did we reach in our study, where we administered only 5 mL/kg of HES in the colloid preload group. To our awareness, safety studies concerning the maximum dose of HES in parturients are lacking but we will agree that volumes in excess of 5 mL/kg may afford no added benefit and therefore we do not suggest exceeding them. Until more studies are performed, adequately powered to evaluate the effect of HES in the population over the long term, we consider that any concern about inducing clinically important coagulopathy in a population already in a hypercoagulable state, such as the parturients investigated in our study, is unsubstantiated.

4. In response to the fourth comment by Drs Akça and Bilotta, we believe that safety issues regarding the fetal effects of HES have been addressed in a number of studies. For instance, in the multicenter CAESAR trial, Mercier et al. measured plasma HES concentrations in venous blood taken from the umbilical cord to assess potential placental transfer. They demonstrated that HES concentration was below the limit of detection in all umbilical cord blood samples, a fact suggesting that placental transfer of HES is insignificant [19]. Additional maternal and neonatal peripartum safety and tolerability data were provided in the CAESAR trial in support of the reassuring use of HES. Similar findings have been observed in the experimental setting, with lack of detectable transplacental transfer in a sheep model [20]. In another study investigating the influence of HES on umbilical cord electrolytes, the authors concluded that the infusion of up to 500 mL of HES does not produce a clinically significant effect on umbilical blood cord electrolytes or on the neonatal acid-base neonatal status [21]. Of note, we did not exceed the volume of 500 mL of HES, since we administered 5 mL/kg as a colloid preload in our study. The insignificant effect of colloid preload or co-load in the obstetric setting has been demonstrated in many studies and corroborated in recent metanalyses and a Cochrane review, which did not show any differences in neonatal acid-base status and Apgar scores between crystalloid and colloid fluid loading techniques [4,11,22]. Finally, a study investigating weight loss among neonates of Cesarean delivery, comparing colloids plus crystalloids versus crystalloids only, showed that the possibility of excess weight loss in the neonates was similar between women exposed to colloids plus crystalloids compared with crystalloids only [23]. This study added confirmatory evidence to the safety profile of both regimes as far as the well-being of the neonate is concerned.

As a final remark, regarding the last comment by Drs Akça and Bilotta about whether it is worth using HES instead of using vasopressors as the first-line therapy in patients undergoing Cesarean delivery, we would like to kindly stress once more that we did not support the sole use of fluids in our study. We compared the incidence of post-spinal anesthesia-induced hypotension in parturients administered either colloid preload or crystalloid co-load in the setting of a prophylactic norepinephrine infusion and we showed that the incidence of hypotension is low and comparable when a norepinephrine infusion is used with either colloid preload or crystalloid co-load. Under no circumstances do we propose the use of either fluid loading technique as a sole intervention, without the use of vasopressors.

We would like to sincerely thank Drs Akça and Bilotta for initiating a fruitful discussion on this important issue. We believe however that the administration of low HES volumes and for a limited period of time during Cesarean section as a supplementary strategy to vasopressors is quite different to its use in the non-obstetric perioperative setting or prolonged administration in intensive care patients, situations that have created safety concerns and led to the recent warnings by regulatory bodies, which we also mentioned in our original study. In any case, new generation colloids such as HES 6% 130/0.4 should be preferred, in view of their superior safety profile.
